# Libman-Sacks Endocarditis As the Initial Presentation of Systemic Lupus Erythematosus With Evolving Multiorgan Sequelae

**DOI:** 10.7759/cureus.114301

**Published:** 2026-08-10

**Authors:** Rafia Ayub, Binuka Gurung

**Affiliations:** 1 Internal Medicine, Manchester University NHS Foundation Trust, Manchester, GBR; 2 Medicine, Manchester University NHS Foundation Trust, Manchester, GBR

**Keywords:** cutaneous lupus erythematous, endocarditis, libman sacks endocarditis (lse), libman-sacks, lupus nephritis (ln), nonbacterial thrombotic endocarditis (nbte), systemic lupus erythematosus

## Abstract

Libman-Sacks endocarditis (LSE) is a rare non-bacterial thrombotic endocarditis (NBTE) characterised by sterile valvular vegetations. It is most associated with systemic lupus erythematosus (SLE) and is often asymptomatic until embolic complications occur, rendering diagnosis challenging.

This report presents the case of a 53-year-old woman initially presenting with abdominal pain secondary to pancreatitis and persistent fevers of unknown origin. Despite extensive investigation, including multiple negative blood cultures, no infectious source was identified. Subsequent transthoracic echocardiography demonstrated a valvular vegetation suggestive of endocarditis, with further assessment producing a clinical diagnosis of the rare LSE subtype. Notably, the patient had no peripheral stigmata of endocarditis, and LSE represented the first manifestation of previously undiagnosed SLE in the context of an underlying connective tissue disorder.

During the hospital course, the patient developed additional manifestations of SLE, including cutaneous lupus erythematosus and lupus nephritis. Autoimmune serology was strongly supportive of SLE. Treatment consisted of corticosteroids, hydroxychloroquine and mycophenolate mofetil, resulting in clinical improvement.

This case is notable as LSE being a first presentation of previously undiagnosed SLE. It highlights the importance of having high suspicion for LSE in patients with culture-negative endocarditis, persistent unexplained fever, and a background of autoimmune disease.

## Introduction

Libman-Sacks endocarditis (LSE) was first described in 1924, following its presentation in four patients with systemic lupus erythematosus (SLE) [1]. It is a form of non-bacterial thrombotic endocarditis (NBTE) characterised by sterile valvular vegetations, most commonly affecting the mitral and aortic valves. Histologically, these vegetations consist of fibrin, neutrophils, lymphocytes, and histiocytes [2].

Common associations of LSE include SLE, antiphospholipid syndrome (APS), and malignancy. It is most strongly associated with SLE, and although likely under-recognised, represents the most characteristic cardiac manifestation of SLE. However, it is novel for LSE to be the first clinical feature of previously undiagnosed SLE, as it was in this case.

Its reported prevalence varies considerably depending on the diagnostic modality used. One cohort study reported a prevalence of LSE of approximately 11% among patients with SLE when diagnosed by transthoracic echocardiography (TTE) [3], whereas post-mortem studies identified valvular involvement in as high as 35-65% of patients with SLE [4]. This discrepancy likely reflects the subclinical nature of the disease.

This asymptomatic nature can make the diagnosis of LSE challenging. It is often clinically silent until complications occur secondary to dislodged emboli from the valvular vegetations, manifesting as transient ischaemic attack (TIA) and stroke. For this reason, it is historically reputed as a post-mortem diagnosis [5]. Diagnosis can also be challenging due to overlap with infective endocarditis (IE), with patients presenting with fever, embolic phenomena and valvular vegetations characteristic of both conditions. Echocardiography remains the cornerstone of investigation, with transoesophageal echocardiography (TOE) offering greater sensitivity than TTE for detecting valvular lesions [4]. Importantly, however, the diagnosis of LSE is largely clinical, as definitive diagnosis is only achievable through histopathological confirmation [1].

Management focuses primarily on treatment of the underlying autoimmune disease and prevention of thromboembolic complications with appropriate anticoagulation. In cases of severe valvular dysfunction or recurrent embolic events not amenable to medical management, surgical intervention such as valvular repair may be required [1].

We present a case of clinically diagnosed LSE as the initial manifestation of previously undiagnosed SLE. The case highlights the diagnostic challenges present when distinguishing LSE from IE in patients who do not have an already established SLE diagnosis and in whom LSE is the first presentation of SLE. This novelty can lead to delayed consideration of LSE, with this case highlighting the importance of earlier autoimmune work-up in patients with culture-negative endocarditis, persistent fever and valvular abnormalities.

## Case presentation

Initial presentation and management

A 53-year-old female first presented to the emergency department with central and left-sided abdominal pain radiating to the back. Investigations demonstrated a raised serum lipase of 666 U/L; abdominal ultrasonography demonstrated a distended gallbladder and computed tomography of the abdomen and pelvis (CT-AP) confirmed acute pancreatitis. Following conservative management and clinical improvement, she was discharged with plans for outpatient elective laparoscopic cholecystectomy.

Two months later, she re-presented with recurrent central and left-sided abdominal pain associated with nausea and persistent fevers. Laboratory findings demonstrated a markedly elevated lipase of 6749 U/L and hypertriglyceridaemia of 25.2 mmol/L (see Table 1). She was admitted under general surgery for acute pancreatitis, thought to be secondary to gallstones and/or raised triglycerides. Initial management consisted of intravenous fluids, variable rate intravenous insulin infusion (VRIII), human albumin solution (HAS), and antibacterial therapy with co-amoxiclav and metronidazole. At this stage, the patient’s persistent fevers were attributed to acute pancreatitis, and no alternative source was considered nor identified.

**Table 1:** Initial laboratory values demonstrating anaemia, an elevated lipid profile, and raised inflammatory markers Acute kidney injury (AKI) grading system: Based on the Acute Kidney Injury Network (AKIN) classification [6], which utilises serum creatinine levels. AKI 1 is defined by an increase in creatinine ≥1.5-2.0 fold from the baseline, AKI 2 is defined by an increase in creatinine ≥2.0-3.0 fold from the baseline, and AKI 3 is defined by an increase in creatinine ≥3.0-fold from the baseline. In this case, an increase from 65 -> 190, equaling a 2.92-fold increase from the baseline creatinine, puts it within the AKI 2 category. '-' denotes there is no reference range for the AKI parameter. All other '-' in the table indicate that no result was taken, either due to haemolysis (in the case of ALT) or due to a lack of repeated testing at this point in the clinical course (in the case of lipase and triglycerides). Bold values should indicate abnormal results.

Blood test	Reference range	Result day 1	Result day 3
White blood cells	4.0-11.0 x 10^9^/L	8.7	7.2
Haemoglobin	115.0-165.0 g/L	104.0	88.0
Mean corpuscular volume (MCV)	80.00-98.00 fL	85.0	82.30
Platelets	150.0-400.0 x 10^9^/L	476.0	277.0
Neutrophils	1.80-7.50 x 10^9^/L	4.78	4.86
Lymphocytes	1.00-4.00 x 10^9^/L	2.99	1.63
Sodium	133-146 mmol/L	138	135
Potassium	3.5-5.3 mmol/L	4.4	4.5
Urea	2.5-7.8 mmol/L	5.1	16.1
Creatinine	45-84 umol/L	65	190
Estimated glomerular filtration rate (eGFR)	>90 mL/min/1.73m^2^	>90	26
Acute kidney injury (AKI)	-	No AKI	AKI 2
Alanine transaminase (ALT)	1-35 IU/L	-	63
Alkaline phosphatase (ALP)	30-130 U/L	97	44
Total bilirubin	0-21 umol/L	8	17
Lipase	13-60 U/L	6749	-
Adjusted calcium	2.20-2.60 mmol/L	2.33	1.90
Phosphate	0.80-1.50 mmol/L	1.22	1.40
C-reactive protein	0-5 mg/L	4	326
Triglycerides	0.0-1.6 mmol/L	25.2	-

During the early stages of her admission, her clinical condition deteriorated. Haemoglobin levels fell acutely, and renal function progressively worsened over the following weeks (see Table 1). Her first input from cardiology came in this initial period due to her profound tachycardia with a raised troponin T of 33 ng/L (reference range <14 ng/L). Electrocardiography (ECG) demonstrated no acute abnormalities, and a repeat troponin was 28 ng/L. Cardiology attributed these findings to physiological stress secondary to her acute illness.

Despite broad-spectrum antibiotic therapy, the patient remained persistently pyrexic. Concern for an intra-abdominal source of infection was raised, and further imaging undertaken. Magnetic resonance cholangiopancreatography (MRCP) showed only a small gallstone within the gallbladder, while repeat CT-AP showed ongoing features of pancreatitis with development of a pancreatic tail pseudocyst, but no evidence of pancreatic necrosis or other convincing intra-abdominal infective focus. The repeat CT imaging did, however, identify a new thrombus within the right portal vein. Following haematology consultation, she was started on therapeutic anticoagulation with dalteparin.

**Figure 1:**
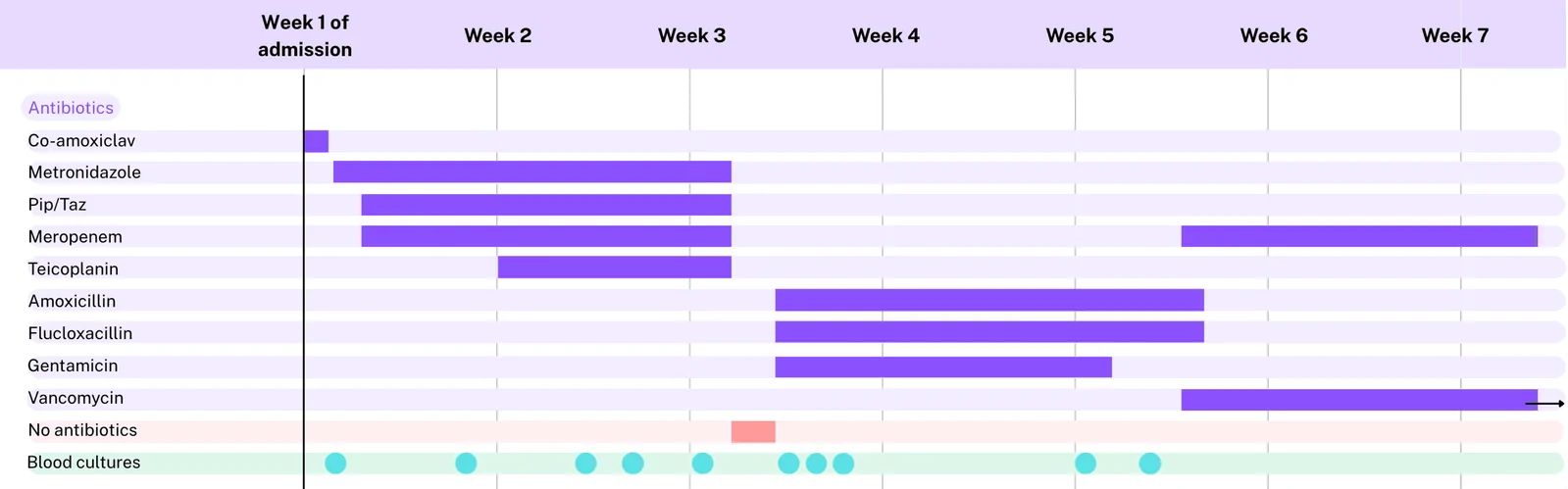
Antibiotic timeline throughout the clinical course Co-amoxiclav: amoxicillin and clavulanic acid; Pip/Taz: piperacillin/tazobactam On the left of this figure are listed all of the antibiotics the patient received during their stay, and the dark purple blocks to the right of each antibiotic demonstrate when during their hospital course they received that particular antibiotic. The figure has a row for the short period of ‘No antibiotics’ that this patient had as an inpatient during week 3 of their stay, represented by a dark red block. The figure has a row outlining when blood cultures were collected during the patient's hospital admission, represented by dark blue dots, plotted with and against the various antibiotics and the short period of time when no antibiotics were received.

As the patient’s fevers persisted, repeated blood cultures remained negative and serial imaging demonstrated no significant changes nor sources of infection. Respiratory viral swabs and sputum cultures were also negative. With ongoing microbiology review, antibiotic therapy was progressively escalated from co-amoxiclav and metronidazole to piperacillin-tazobactam, and subsequently to meropenem and teicoplanin (the antibiotic prescription course in its completion can be viewed in Figure 1). Despite this, the patient remained persistently febrile. Additional investigations were done, including procalcitonin and beta-D-glucan testing, which were also negative, further increasing the diagnostic uncertainty associated with this case.

Diagnosis of endocarditis

Given the patient’s persistent fevers despite escalation of broad-spectrum antibiotic therapy, and the absence of an identifiable infection source to explain this, microbiology recommended assessment for IE, including clinical examination for peripheral stigmata and TTE. The patient denied chest pain, had no audible cardiac murmurs, and no peripheral stigmata of IE on examination.

**Figure 2:**
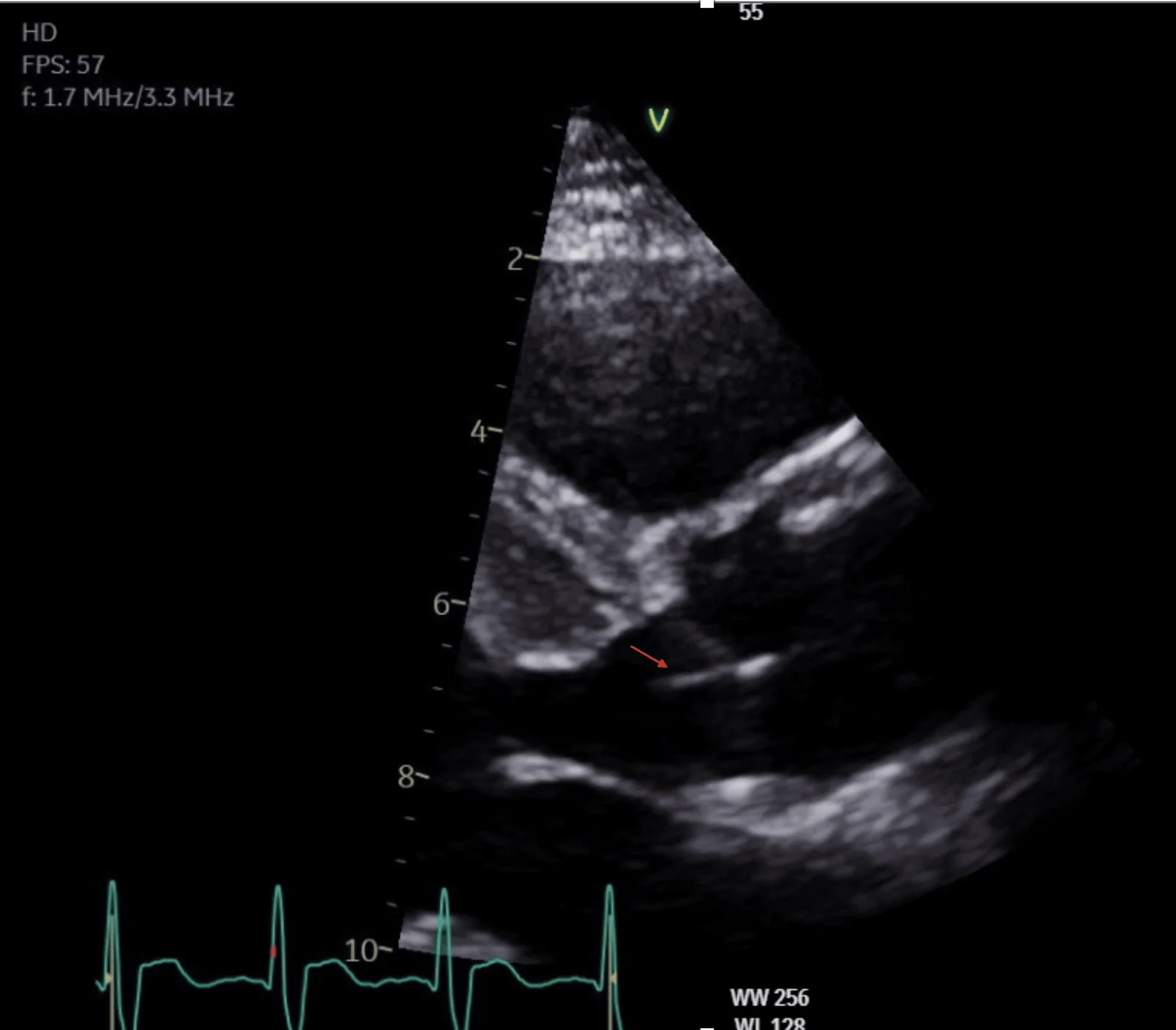
Transoesophageal echocardiography (TOE) findings: Focused parasternal long-axis view at the level of the aortic valve demonstrating a small, mobile, echogenic structure attached to the aortic valve The red arrow points to the small, mobile, echogenic structure attached to the aortic valve. This supported a diagnosis of endocarditis, although it was unclear whether this was infective or non-infective.

TTE demonstrated a small mobile echogenic structure on the aortic valve, visualised only in the parasternal long axis view (see Figure 2 and Videos 1-2). IE could not be fully excluded from this alone. In the context of persistently negative blood cultures, cardiology considered culture-negative fastidious Gram-negative bacteria - *Haemophilus*, *Aggregatibacter*, *Cardiobacterium*, *Eikenella*, *Kingella* (HACEK) - endocarditis as the most likely diagnosis at this point in the case. Prolonged blood culture incubation was requested to investigate HACEK species. Empirical treatment with amoxicillin, flucloxacillin and gentamicin was started, and a TOE was requested to further characterise the valvular lesion. The patient was transferred under the care of the infectious diseases team.

**Video 1:** Transthoracic echocardiography (TTE) findings demonstrating a small, mobile, echogenic structure attached to the aortic valve

**Video 2:** Transthoracic echocardiography (TTE) findings: Doppler echocardiogram obtained in the parasternal view, demonstrating mild aortic regurgitation secondary to the echogenic structure attached to the aortic valve

Whilst awaiting TOE, the patient developed an acute left-sided paralysis. Urgent CT angiography and MRI of the brain were performed, which showed no acute arterial occlusion; however, cortical laminar necrosis was identified, and an embolic aetiology could not be excluded. Stroke and neurology teams reviewed the case, concluding that the cortical infarctions were most likely embolic in origin, especially given the right portal vein thrombosis already identified. The patient had a history of thrombotic thrombocytopenic purpura (TTP) and sickle cell disease, deemed unrelated to these findings. Therapeutic dalteparin (already commenced due to the right portal vein thrombosis) was continued. Arrangements were made for follow-up in the TIA clinic following discharge. This occurrence of an apparent embolic cerebral infarction furthered the concern that the valvular vegetative lesion represented a clinically significant endocarditis.

Rheumatology review

At this stage, an inpatient rheumatology review was requested due to the patient’s persistent culture-negative fevers and new embolic stroke, raising concern for an underlying autoimmune or vasculitis process. The patient had a significant rheumatological background, including secondary Raynaud’s phenomenon and a previously suspected mixed connective tissue disease (MCTD) with no formal associated diagnosis. Previous serological investigations prior to her admission demonstrated multiple positive autoimmune markers, including antinuclear antibody (ANA), anti-Sjogren's-syndrome type A and B antibodies (SSA, SSB), ribonucleoprotein antibody (RNP), anti-smooth muscle antibody (Anti-Sm), and chromatin antibodies, in support of an underlying MCTD. Cardiolipin and lupus anticoagulant were negative.

Following review, rheumatology advised that an active autoimmune disease could be contributing to the current presentation, with SLE considered a possible unifying diagnosis; this marked the first point at which active SLE was considered a diagnostic association in this patient’s case. Further autoimmune investigations were requested (see Table 2), including complement (C3/C4) levels, immunoglobulin profile and tests for various antibodies. A Crithidia assay was also performed, a specific blood test used to detect anti-double stranded DNA (anti-dsDNA) antibodies, which subsequently returned negative. Given the high suspicion of active autoimmune disease, the patient was initiated on hydroxychloroquine and prednisolone.

**Table 2:** Autoimmune serology for the workup of an underlying autoimmune pathology Anti-SSA: anti-Sjogren's-syndrome type A antibody, also known as anti-Ro; anti-SSB: anti-Sjögren's syndrome type B antibody, also known as anti-La; anti-HisRS: anti-histidyl-tRNA synthetase antibody, also known as anti-Jo-1

Autoimmune serology	Result
Antinuclear antibody (ANA)	Positive
Anti-SSA (Ro) antibody	Positive
Anti-SSB (La) antibody	Positive
Anti-RNP (ribonucleoprotein) antibody	Positive
Anti-smooth muscle (anti-Sm) antibody	Positive
Anti-chromatin antibody	Positive
Ribonucleoprotein (RNP) 68 antibody	Positive
Anti-HisRS (Jo-1) antibody	Negative
Scleroderma-70 (Scl-70) antibody	Negative
Immunoglobulin G (IgG) double-stranded DNA (dsDNA)	Negative
Anti-cardiolipin antibody	Negative
Beta-2 (B2) glycoprotein 1 antibody	Negative
Complement - component 3 (C3) and component 4 (C4)	Negative
Crithidia test	Negative
Lupus anticoagulant	Negative
Myositis extractable nuclear antigen (ENA)	Negative

**Figure 3:**
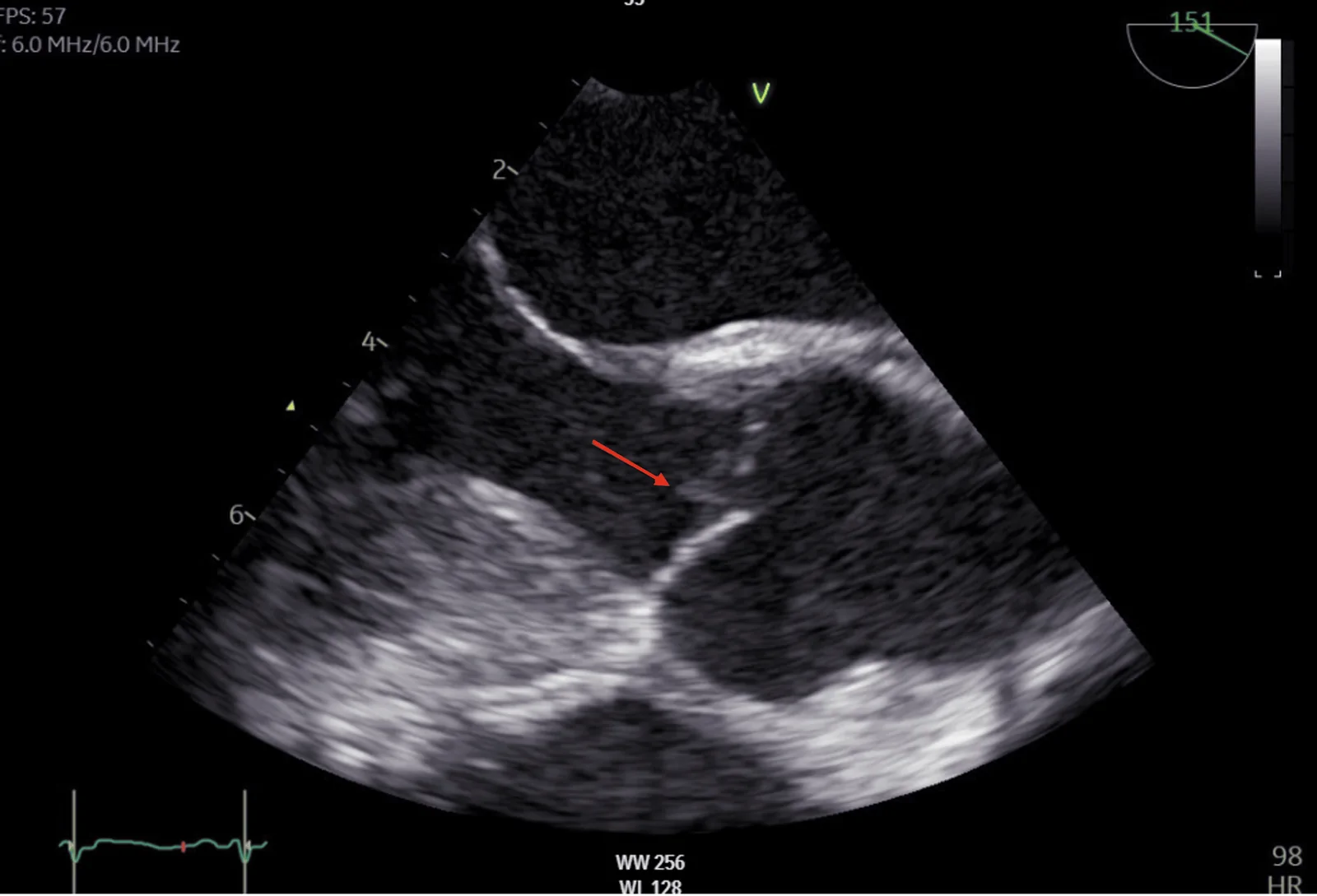
Transoesophageal echocardiography (TOE) findings: still image from the TOE demonstrating a 9 × 7 mm echogenic structure attached to the right coronary cusp of the aortic valve The red arrow points to the 9 × 7 mm echogenic structure attached to the aortic valve, which appears grey on the image.

TOE demonstrated a 9 x 7 mm echogenic structure attached to the right coronary cusp of the aortic valve, with associated irregularity of the aortic root, further supporting an endocarditis diagnosis (see Figure 3). Antimicrobial therapy was therefore continued. A CT aortogram was subsequently performed for further characterisation of the vegetation but demonstrated no additional significant abnormalities, and no aortic root abscess. Despite ongoing treatment, however, the patient continued to have persistent fevers. Antimicrobial therapy was escalated further to meropenem and vancomycin.

Dermatological manifestations

**Figure 4:**
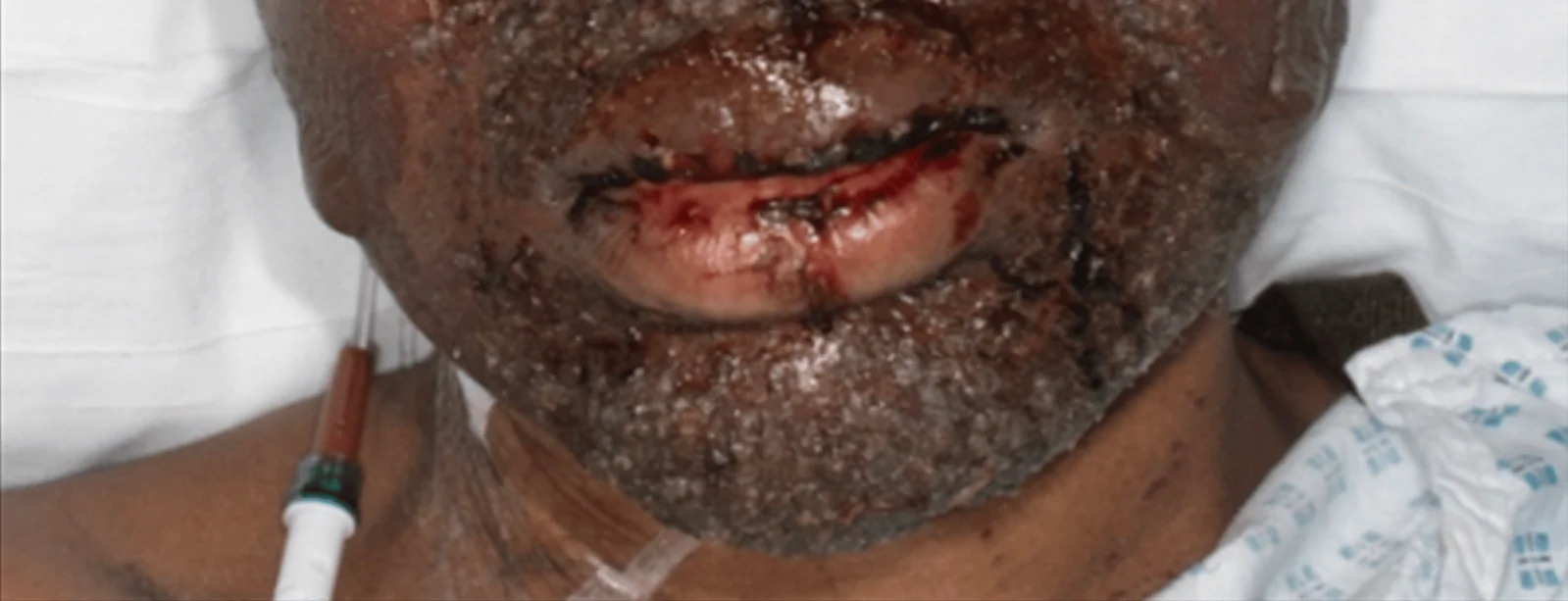
Facial rash characterised by vesicular-bullous lesions across sun-exposed regions of the face, worst surrounding the mouth and jawline; the appearance aligns with cutaneous lupus erythematosus

At this stage in the hospital course, the patient developed a new facial rash. Initially erythematous, the eruption progressed to a vesicular-bullous rash affecting sun-exposed areas of the face only. Although not confined to a classical malar distribution, the rash was most prominent over both cheeks, where large bullous lesions were present on an erythematous base. The nose was relatively spared. The chin was affected with crusting extending from the jawline onto the neck. A few erythematous low-grade inflammatory lesions were present across the lips, although the buccal mucosa was spared (see Figure 4). There was no involvement of the sclerae or conjunctivae. Post-inflammatory macules were present on the chest wall, along with post-inflammatory changes affecting the palmar aspects of the digits.

Following review, both dermatology and rheumatology considered the rash to be most consistent with cutaneous lupus erythematosus in the context of an evolving systemic autoimmune process. A skin biopsy was subsequently requested to further characterise the eruption.

IE multi-disciplinary team (MDT) discussion outcome

The case was discussed at the IE MDT meeting, where three possible causes of the vegetation were discussed. These included bacterial vegetation suggestive of an IE, embolic phenomenon and autoimmune phenomenon, i.e., Libman-Sacks. IE MDT recommended that, following reviewing imaging and other aspects of the patient’s clinical history, including deteriorating renal function, new rash, and extensive autoimmune background, the findings were considered more consistent with SLE-associated LSE; this was a clinical diagnosis, given the lack of histopathological confirmation. This was the likely cause of the temperature spikes, so all antibiotics for endocarditis were finally stopped. The patient was continued on medication and treatments as per the rheumatology team and followed up accordingly.

Long-term speciality follow-up

Rheumatology Input

The patient was started on hydroxychloroquine and prednisolone during her admission. As an outpatient, she was started on mycophenolate, and her prednisolone was slowly weaned. At six months post-discharge, the Systemic Lupus Erythematosus Disease Activity Index (SLEDAI) was calculated; this is a validated model of disease activity in lupus, monitoring for SLE disease activity across nine organ systems [7]. She scored 22, indicating high disease activity according to the SLEDAI criteria.

Cardiology Input and Follow-Up Echocardiograms

Following the patient’s discharge in March, they had further echocardiograms to monitor their vegetative lesion - the first one month post-discharge, and the second six months post-discharge. These continued to demonstrate the lesion, although aortic regurgitation fully resolved (see Video 3).

**Video 3:** Echocardiography (ECHO) findings after six months: The echogenic structure on the aortic valve is almost entirely resolved, with no aortic regurgitation

Dermatology Input

The management for the patient’s rash included topical tacrolimus (0.3%) combined with Dermovate for 14 days, followed by tacrolimus (0.1%) until healed. Emollient therapy with 50/50 liquid paraffin (LP) and white soft paraffin (WSP) ointment was also advised. A skin biopsy of the rash behind the ear was also obtained, where histopathology showed non-specific features consistent with either cutaneous lupus or connective tissue disease. The relatively mild histological changes were felt by dermatology to reflect a partial treatment response, as the patient had clinically improved and was already receiving systemic corticosteroids at the time of biopsy. Maintenance topical therapy included daily high-factor (SPF 50) sun protection.

Other Speciality Input

Given the patient's multi-organ involvement, typical of SLE, they received further speciality follow-up. For the portal vein thrombosis, haematology advised completion of three months of therapeutic anticoagulation, for the clot likely being provoked secondary to endocarditis and inflammatory disease. Follow-up imaging demonstrated complete resolution of the thrombus. During the patient's hospital admission, they also developed worsening renal function with elevated urine protein-creatinine ratio and recurrent hyperkalaemia. When considering her evolving systemic presentation, this was consistent with renal involvement secondary to SLE, or lupus nephritis. Nephrology review recommended close monitoring of renal function, with consideration of renal biopsy should renal function decline or the patient develop haematuria or proteinuria. However, on follow-up a few months after her hospital admission, the patient reached a state of complete renal response with stable kidney function (glomerular filtration rate (GFR) 54 mL/min/1.73m^2^), and biopsy was not needed.

## Discussion

LSE as first presentation of SLE

This case presents a rare form of endocarditis, LSE, clinically diagnosed as a first manifestation of SLE, and resulting in SLE sequelae requiring extensive follow-up. Although LSE is often considered rare, its true prevalence may be underestimated due to its usually asymptomatic nature with reliance on imaging modalities for detection [3,4]. Therefore, the diagnosis should not be disregarded, particularly in patients presenting with culture-negative valvular abnormalities and an autoimmune background. A similar case reported by Saulat et al. [8] described LSE also as a presenting feature of SLE and other autoimmune conditions, emphasising the need for increased clinical awareness and consideration of the condition.

Challenges to diagnosis

Several factors contributed to the delayed clinical diagnosis of LSE in this case. Firstly, the patient’s initial presentation of gallstone pancreatitis. During early stages of the hospital course, the patient’s pyrexia was attributed to pancreatitis, potentially leading to "premature closure" [9]. Retrospectively, this may have delayed consideration of alternative causes of fever such as endocarditis. Following rheumatological review, the pancreatitis was felt to be entirely unrelated to the SLE diagnosis, and more likely secondary to hypertriglyceridemia.

Secondly, distinguishing non-infective LSE from IE proved challenging. Although three sets of blood cultures remained negative, the patient’s persistent pyrexia maintained clinical concern for IE, particularly as the patient initially lacked other overt manifestations of active SLE. Given the potential negative consequences of untreated IE, management continued the assumption of infection until there was sufficient evidence to support an alternative non-infective diagnosis.

Diagnostic uncertainty persisted due to conflicting findings within the diagnostic framework for IE. For a diagnosis to be made under the modified Duke criteria, either two major, one major and three minor, or five minor criteria must be met [10]. The patient met one major criterion (echocardiographic evidence of vegetation) and one minor criterion (fever) but lacked further evidence of infection (e.g. microbiological evidence such as positive blood cultures) and demonstrated no peripheral stigmata of IE. Consequently, an IE diagnosis could not be established on Duke criteria alone.

Reasons for the endocarditis being culture-negative had to be considered. This was what prompted investigation for HACEK organisms, which do not show up well on standard blood cultures given their fastidious and slow-growing nature, necessitating prolonged cultures [11]. Another consideration was the timing of blood culture draws compared with antibiotic use leading to possible false-negative cultures, outlined further in the Discussion*.*

Eventual LSE diagnosis was clinical due to a lack of valvular histopathology, requiring careful consideration of all clinical features in this case. It was only through review of the TOE findings, multidisciplinary endocarditis team input, and the evolving clinical picture riddled with SLE sequelae that LSE could be clinically diagnosed.

Previous rheumatology history

Another notable aspect in this case was the patient’s significant autoimmune history; she had a diagnosis of mixed connective tissue disorder and medically managed secondary Raynaud’s disease, for which she was already under rheumatology care. Additionally, she had multiple positive autoimmune antibodies and a history of TTP. Taken together, this clinical background, serological findings and TTE findings all supported the eventual clinical diagnosis of LSE associated with SLE.

Other than the patient’s autoimmune history and positive autoimmune serology, their development of additional clinical manifestations of active SLE such as cutaneous involvement and lupus nephritis were all significant. However, these features emerged gradually during the hospital course, only later forming a recognisable autoimmune clinical sequela. Earlier development of these manifestations may have prompted consideration of SLE-associated LSE sooner, highlighting the diagnostic challenge posed by LSE when it presents before other overt manifestations of SLE. A final factor more supportive of an underlying autoimmune pathology was failure to respond to prolonged, broad-spectrum antibiotics, and improvement only after high-dose corticosteroids and immunosuppressive therapy - the cornerstones of SLE treatment [12]. Although the clinical diagnosis of LSE feels appropriate given all these factors, this case emphasises the importance of maintaining a broad differential diagnosis in patients with culture-negative endocarditis, especially in patients with underlying autoimmune disease.

Possible false-negative blood cultures

Multiple blood cultures were taken, as shown in Figure 1 earlier in the case, all of which were negative. However, there were no significant periods of antibiotic cessation throughout their hospital course, meaning these cultures were all obtained whilst the patient remained on antimicrobial therapy. This was due to the patient’s persistent fevers and ongoing clinical deterioration; although the standard is to withhold antibiotics for approximately 72 hours prior to blood culture collection and thereby reduce false-negative results [13], this applies only when considered clinically safe. In this case, there was significant concern that even a brief period of antibiotic discontinuation could risk progression of an underlying infection of which no source had yet been identified.

The clinical diagnosis was ultimately most consistent with LSE, which is non-infective, and therefore the antibiotics were unlikely to have provided any therapeutic benefit. Nevertheless, this case highlights the challenging balance between optimising diagnostic accuracy and ensuring patient safety. The perceived need to maintain antimicrobial coverage whilst diagnosis remained uncertain superseded the benefit of obtaining blood cultures following an antibiotic-free period. Determining when it is safe to prioritise diagnostic accuracy over empirical antibiotic therapy remains a complex clinical judgement, especially in cases of suspected endocarditis.

## Conclusions

Although uncommon, this case demonstrates LSE as an initial presentation of SLE, even in the absence of a previously established SLE diagnosis. The diagnosis of LSE can be particularly challenging when patients present with persistent fever, valvular vegetations and embolic phenomena, as these features can closely mimic IE. Consequently, LSE should be considered as a possible diagnosis in cases of culture-negative endocarditis that do not fulfil Duke’s criteria, in patients with underlying autoimmune disease or positive autoimmune serology, even before classical features of SLE have become apparent.

Early recognition is also important given the potential for significant morbidity from embolic complications, and the marked difference in therapeutic strategies for inflammatory as opposed to IE. Establishing a clinical diagnosis rather than a histopathological one often requires extensive, time-consuming work-up with integration of clinical, microbiological, serological and echocardiographic findings, with multidisciplinary collaboration. This case highlights the importance of maintaining a broad differential diagnosis in culture-negative endocarditis, as timely recognition of LSE can lead to appropriate immunosuppressive therapy and improved patient outcomes.

## References

[1] Ibrahim AM, Siddique MS (2023). Libman-Sacks endocarditis. StatPearls [Internet].

[2] Doherty NE, Siegel RJ (1985). Cardiovascular manifestations of systemic lupus erythematosus. Am Heart J.

[3] Roldan CA, Tolstrup K, Macias L, Qualls CR, Maynard D, Charlton G, Sibbitt WL Jr (2015). Libman-Sacks endocarditis: detection, characterization, and clinical correlates by three-dimensional transesophageal echocardiography. J Am Soc Echocardiogr.

[4] Hojnik M, George J, Ziporen L, Shoenfeld Y (1996). Heart valve involvement (Libman-Sacks endocarditis) in the antiphospholipid syndrome. Circulation.

[5] Yoo BW, Lee SW, Song JJ, Park YB, Jung SM (2020). Clinical characteristics and long-term outcomes of Libman-Sacks endocarditis in patients with systemic lupus erythematosus. Lupus.

[6] Mehta RL, Kellum JA, Shah SV, Molitoris BA, Ronco C, Warnock DG, Levin A (2007). Acute Kidney Injury Network: report of an initiative to improve outcomes in acute kidney injury. Crit Care.

[7] Bombardier C, Gladman DD, Urowitz MB, Caron D, Chang CH (1992). Derivation of the SLEDAI. A disease activity index for lupus patients. The Committee on Prognosis Studies in SLE. Arthritis Rheum.

[8] Saulat SR, AlSolami AL, Gabr YS (2026). Libman-Sacks endocarditis as the initial presentation of systemic lupus erythematosus and antiphospholipid syndrome: a multisystem diagnostic challenge. Cureus.

[9] Kumar B, Kanna B, Kumar S (2011). The pitfalls of premature closure: clinical decision-making in a case of aortic dissection. BMJ Case Rep.

[10] Fowler VG, Durack DT, Selton-Suty C (2023). The 2023 Duke-International Society for Cardiovascular Infectious Diseases criteria for infective endocarditis: updating the modified Duke criteria. Clin Infect Dis.

[11] Wassef N, Rizkalla E, Shaukat N, Sluka M (2013). HACEK-induced endocarditis. BMJ Case Rep.

[12] Katarzyna PB, Wiktor S, Ewa D, Piotr L (2023). Current treatment of systemic lupus erythematosus: a clinician's perspective. Rheumatol Int.

[13] Horstkotte D, Follath F, Gutschik E (2004). Guidelines on prevention, diagnosis and treatment of infective endocarditis executive summary; the task force on infective endocarditis of the European society of cardiology. Eur Heart J.

